# Supplementary material for: Insulin regulates POMC neuronal plasticity to control glucose metabolism
Source: eLife. 2018 Sep 19;7:e38704. doi: 10.7554/eLife.38704 (PMC6170188; doi:10.7554/eLife.38704)
Supplement: Supplementary file 1. — Statistical significance and P values (Yate’s continuity corrected Chi-squared test) comparing populations of POMC neurons that are activated, inhibited or unresponsive to insulin in Ptpn2fl/fl, POMC-TC and POMC-TC-IR mice. [file elife-38704-supp1.docx]

| **Chi-squared test** | **Significance** | **P Value** |
| --- | --- | --- |
| **Excited** |  |  |
| Control vs POMC-TC | *** | <0.001 |
| POMC-TC vs POMC-TC-IR | ** | 0.0039 |
| Control vs POMC-TC-IR | ns | 0.3 |
| **Inhibited** |  |  |
| Control vs POMC-TC | * | 0.046 |
| POMC-TC vs POMC-TC-IR | ns | 0.37 |
| Control vs POMC-TC-IR | ** | 0.0024 |
| **Non-Responsive** |  |  |
| Control vs POMC-TC | ns | 0.13 |
| POMC-TC vs POMC-TC-IR | *** | <0.001 |
| Control vs POMC-TC-IR | * | 0.033 |

* = P value <0.05, ** = P value <0.01, *** = P value <0.001, ns = non-significant
