# Supplementary material for: Insulin regulates POMC neuronal plasticity to control glucose metabolism
Source: eLife. 2018 Sep 19;7:e38704. doi: 10.7554/eLife.38704 (PMC6170188; doi:10.7554/eLife.38704)
Supplement: Supplementary file 2. — Statistical significance and P values (Yate’s continuity corrected Chi-squared test) comparing populations of POMC neurons from chow or high fat fed (HFF) mice that are activated, inhibited or unresponsive to insulin after administration of vehicle or TCPTP inhibitor (compound 8, 20 nM). [file elife-38704-supp2.docx]

| **Chi-squared test** | **Significance** | **P Value** |
| --- | --- | --- |
| **Excited** |  |  |
| Control vs TCPTP Inhibitor | *** | 0.003 |
| Chow vs HFF | * | 0.0266 |
| HFF vs HFF+TCPTP Inhibitor | *** | <0.001 |
| **Inhibited** |  |  |
| Control vs TCPTP Inhibitor | *** | <0.001 |
| Chow vs HFF | * | 0.0449 |
| HFF vs HFF+TCPTP Inhibitor | *** | <0.001 |
| **Non-Responsive** |  |  |
| Control vs TCPTP Inhibitor | * | 0.0233 |
| Chow vs HFF | ns | 0.3737 |
| HFF vs HFF+TCPTP Inhibitor | ns | 0.1441 |

* = P value <0.05, ** = P value <0.01, *** = P value <0.001, ns = non-significant
