# Supplementary material for: Insulin regulates POMC neuronal plasticity to control glucose metabolism
Source: eLife. 2018 Sep 19;7:e38704. doi: 10.7554/eLife.38704 (PMC6170188; doi:10.7554/eLife.38704)
Supplement: Transparent reporting form [file elife-38704-transrepform.docx]

***eLife’s* transparent reporting form**

We encourage authors to provide detailed information *within their submission* to facilitate the interpretation and replication of experiments. Authors can upload supporting documentation to indicate the use of appropriate reporting guidelines for health-related research (see [EQUATOR Network](http://www.equator-network.org/%20)), life science research (see the [BioSharing Information Resource](https://biosharing.org/)), or the [ARRIVE guidelines](http://www.plosbiology.org/article/info:doi/10.1371/journal.pbio.1000412) for reporting work involving animal research. Where applicable, authors should refer to any relevant reporting standards documents in this form.

If you have any questions, please consult our Journal Policies and/or contact us: [editorial@elifesciences.org](mailto:editorial@elifesciences.org).

**Sample-size estimation**

- You should state whether an appropriate sample size was computed when the study was being designed
- You should state the statistical method of sample size computation and any required assumptions
- If no explicit power analysis was used, you should describe how you decided what sample (replicate) size (number) to use

Please outline where this information can be found within the submission (e.g., sections or figure legends), or explain why this information doesn’t apply to your submission:

Sample size was based upon prior experience and preliminary data.

To determine the effects of *Ptpn2* deletion in POMC neurons upon whole-body glucose metabolism, experimental sample size was based upon preliminary hyperinsulinemic euglycemic clamp experiments between 10-week old male *Ptpn2^fl/fl^* vs *Pomc*-Cre *Ptpn2^fl/fl^*. In this experiment the rate of glucose appearance during the clamp (a readout of hepatic gluconeogenesis) was: *Ptpn2^fl/fl^* mean = 0.29 ± 0.45 mg/kg/min vs *Pomc*-Cre *Ptpn2^fl/fl^* mean = 5.2 ± 1.8 mg/kg/min. Based on these data, a sample size of 5 will be required to detect differences between treatments (power 80%, two-sided α=0.01).

To determine the effects of *Ptpn2* deletion in POMC neurons upon neuronal activation, experimental sample size was based upon preliminary functional immunohistochemical experiments between 8-week old male *Pomc*-eGFP in response to vehicle or insulin. In this experiment then number of POMC neuons that co-expressed c-Fos was: *Pomc*-eGFP + Vehicle mean = 21 ± 9 activated POMC cells vs *Pomc*-eGFP + Insulin (0.85mU/g, i.p.) 86 ± 17 activated POMC cells. Based on these data, a sample size of 4 will be required to detect differences between treatments (power 80%, two-sided α=0.01).

**Replicates**

- You should report how often each experiment was performed
- You should include a definition of biological versus technical replication
- The data obtained should be provided and sufficient information should be provided to indicate the number of independent biological and/or technical replicates
- If you encountered any outliers, you should describe how these were handled
- Criteria for exclusion/inclusion of data should be clearly stated
- High-throughput sequence data should be uploaded before submission, with a private link for reviewers provided (these are available from both GEO and ArrayExpress)

Please outline where this information can be found within the submission (e.g., sections or figure legends), or explain why this information doesn’t apply to your submission:

All *n* numbers can be founded embedded within each figure or figure legend. The number of independent biological replicates is also stated within the figure legend where appropriate.

**Statistical reporting**

- Statistical analysis methods should be described and justified
- Raw data should be presented in figures whenever informative to do so (typically when N per group is less than 10)
- For each experiment, you should identify the statistical tests used, exact values of N, definitions of center, methods of multiple test correction, and dispersion and precision measures (e.g., mean, median, SD, SEM, confidence intervals; and, for the major substantive results, a measure of effect size (e.g., Pearson's r, Cohen's d)
- Report exact p-values wherever possible alongside the summary statistics and 95% confidence intervals. These should be reported for all key questions and not only when the p-value is less than 0.05.

Please outline where this information can be found within the submission (e.g., sections or figure legends), or explain why this information doesn’t apply to your submission:

All statistical analysis methods and information regarding statistical tests, n values, dispersion and precision measurements are described in the figures, figure legends and methods section.

(For large datasets, or papers with a very large number of statistical tests, you may upload a single table file with tests, Ns, etc., with reference to sections in the manuscript.)

**Group allocation**

- Indicate how samples were allocated into experimental groups (in the case of clinical studies, please specify allocation to treatment method); if randomization was used, please also state if restricted randomization was applied
- Indicate if masking was used during group allocation, data collection and/or data analysis

Please outline where this information can be found within the submission (e.g., sections or figure legends), or explain why this information doesn’t apply to your submission:

Mice were all randomly allocated to treatment groups and no masking was used during group allocation, data collection and/or data analysis.

**Additional data files (“source data”)**

- We encourage you to upload relevant additional data files, such as numerical data that are represented as a graph in a figure, or as a summary table
- Where provided, these should be in the most useful format, and they can be uploaded as “Source data” files linked to a main figure or table
- Include model definition files including the full list of parameters used
- Include code used for data analysis (e.g., R, MatLab)
- Avoid stating that data files are “available upon request”

Please indicate the figures or tables for which source data files have been provided:

0
